# Psychometric validation of the Dysphagia Symptom Questionnaire in patients with eosinophilic esophagitis treated with budesonide oral suspension

**DOI:** 10.1186/s41687-017-0006-5

**Published:** 2017-09-12

**Authors:** Stacie Hudgens, Christopher Evans, Elaine Phillips, Malcolm Hill

**Affiliations:** 1 Clinical Outcome Solutions, 3709 North Campbell, Tucson, AZ 85719 USA; 2 Endpoint Outcomes, 11 Beacon Street, Suite 910, Boston, MA 02108 USA; 3 Formerly at Meritage Pharma, Inc., 12555 High Bluff Drive #385, San Diego, CA 92130 USA

**Keywords:** Budesonide oral suspension, Dysphagia, Eosinophilic esophagitis, Minimal clinically important difference, Patient-reported outcome, Psychometric properties, Responsiveness, Validity

## Abstract

**Background:**

Eosinophilic esophagitis (EoE) is characterized by high levels of eosinophils in the esophageal mucosa. Patients with the disease present with a range of symptoms, including dysphagia (difficulty swallowing). The aim of this analysis was to assess the psychometric properties of the Dysphagia Symptom Questionnaire (DSQ), a patient-reported outcome (PRO) measure of dysphagia associated with EoE. Psychometric properties of the DSQ were assessed using data collected from a 12-week, phase 2, multicenter, randomized, double-blind, placebo-controlled trial of budesonide oral suspension in adolescents and adults (11–40 years old) with EoE.

**Results:**

The study population comprised 93 patients with EoE; 94.6% of whom were white, 68.8% were male and the mean age (standard deviation) was 21.6 (7.7) years. Patients had been diagnosed with EoE for a mean of 37.6 months before study initiation. The DSQ was feasible to implement with few item-level data missing at baseline. Item discrimination was high, with floor and ceiling effects below the predefined threshold (≤9%). Higher DSQ scores corresponded with presence and increased severity of dysphagia, indicative of strong item discrimination among patients at baseline (threshold >50%). The DSQ was able to detect changes in symptoms over time and produced similar outcomes to those from physician- and other patient-rated measures, supportive of construct validity. The DSQ had strong test–retest reliability (intraclass correlation coefficient, *r* = 0.82); and was also responsive to disease-level changes, with higher DSQ scores corresponding to increased esophageal eosinophilic burden. Lastly, the percentage changes in the minimal clinically important difference and clinically important difference in DSQ score were estimated at −27.4% and −55.4%, respectively.

**Conclusions:**

These analyses support the DSQ as a valid and reliable measure of dysphagia in patients with EoE. Changes in DSQ scores suggest a level of agreement between clinician, patient and histologic response. The DSQ should therefore be considered a viable PRO measure of dysphagia for use in future therapeutic studies of EoE.

## Background

Eosinophilic esophagitis (EoE) is a relatively newly described disease [1], and is defined as a chronic, allergen- and immune-mediated condition characterized by esophageal dysfunction with unusually high levels of eosinophilic infiltration of the esophageal mucosa (≥15 eosinophils/high-power field [eos/hpf]) [2]. Dysphagia (difficulty swallowing) and food impaction are the most common symptoms in adolescents and adults presenting with EoE [2]. In children, other symptoms, such as feeding intolerance, feeding refusal, heartburn and chest pain, are more common [2]. With increasing EoE disease duration and age, there is a concomitant and significant increase in dysphagia symptoms, esophageal strictures and risk of food impaction requiring interventional measures [3–5]. A recent meta-analysis of North American and European population-based studies of patients with EoE has estimated that the prevalence and incidence of this disease has been steadily increasing over time [6], with the healthcare-related cost of EoE estimated to be as high as $1.36 billion per annum in the USA (data collected from 1 January 2009 to 31 December 2010) [7].

The consensus recommendations for EoE advise that topical corticosteroid therapy be considered for children and adults after initial diagnosis and for the long-term maintenance of symptoms [8]; however, no therapies are currently approved by the US Food and Drug Administration (FDA) or the European Medicines Agency for this indication. Budesonide oral suspension (BOS), a novel muco-adherent, topical corticosteroid formulation designed specifically for the treatment of EoE, has been shown to induce a histologic response in children with this disease [9]. A recent phase 2 trial has also shown that treatment with BOS improves dysphagia, histologic and endoscopic features in adolescents and adults with EoE (ClinicalTrials.gov identifier: NCT01642212) [10, 11]. The ability to accurately quantify dysphagia symptoms in patients with EoE, using a validated patient-reported outcome (PRO) measure, would aid the development of new pharmacologic agents, such as BOS, for the treatment of EoE.

The aim of this analysis was to assess the psychometric properties of the Dysphagia Symptom Questionnaire (DSQ), a PRO symptom measure designed to specifically assess dysphagia frequency and severity in patients with EoE, using data from a phase 2 trial of BOS (ClinicalTrials.gov identifier: NCT01642212). The content validity of the DSQ is reported elsewhere [12].

## Methods

### Study design and study population

The psychometric properties of the DSQ were assessed using data collected from a 12-week, phase 2, multicenter, randomized, double-blind, placebo-controlled, parallel-arm trial of BOS; ClinicalTrials.gov identifier: NCT01642212 [10], which enrolled patients with EoE aged 11–40 years. The lower bound of this range was selected as the minimum age at which patients would be able to self-assess dysphagia and use the DSQ. The upper bound of this range was selected because older patients are more likely to develop fibrostenotic disease, which is typically refractory to anti-inflammatory treatment. Patients participated in a 6-week screening period, during which the DSQ was completed daily on an electronic hand-held device for at least 3 out of the 6 weeks. Patients with sufficient dysphagia (a minimum of 4 days with dysphagia and ≥70% compliance with the DSQ within any 2 of the first 3 weeks of screening) entered a 4-week, single-blind placebo run-in (baseline) period during which they received placebo twice daily and completed the DSQ once per day. The baseline period consisted of two visits: visit 1 (start of baseline period); and visit 2 (end of baseline period ±2 days). Patients who fulfilled the baseline eligibility criteria were randomized 1:1 to receive BOS (2.0 mg/10 mL) or placebo (10 mL) twice daily for 12 weeks, during which they were asked to complete the DSQ once per day. The inclusion criteria for the randomized phase included histologic evidence of EoE (≥15 eos/hpf) from at least two levels of the esophagus within 10 weeks of the baseline period; a minimum of 4 days with dysphagia within 2 weeks of randomization; acceptable diary compliance (≥70% of days within 2 weeks of randomization); and ≥70% compliance with placebo at randomization.

### The Dysphagia Symptom Questionnaire

The DSQ was developed specifically to measure dysphagia associated with EoE [12]. The DSQ (version 4.0) uses a daily recall period and comprises three questions on the presence and severity of EoE dysphagia (questions 1–3) (Table 1). All patients respond to questions 1 and 2, and are required to have eaten solid foods (‘Yes’ to question 1) in order to proceed with the questionnaire. Patients who respond ‘No’ to question 2 are given a score of zero, and do not go on to answer question 3 (the diary is recorded as completed for that day). Those who respond ‘Yes’ to questions 1 and 2 move on to question 3, which is scored on a five-point scale that infers severity of dysphagia based on the patient’s action to alleviate symptoms, ranging from no action to seeking medical attention. Question 1 was not included in the scoring algorithm on the severity of dysphagia, as the incidence of solid food avoidance is thought to be low in patients with EoE [12]. In addition, if a patient answered ‘No’ to question 1, the remaining items on the DSQ were not scored. Although question 1 was not scored, the proportion of patients who reported avoiding solid foods is captured in the binary response to question 1 (response = ‘No’). A fourth question on pain related to dysphagia was included as a standalone item on the DSQ (Table 1). From interviews with adolescent and adult patients with EoE, pain was not highlighted as an important symptomatic factor and question 4 was thus considered an exploratory item and not included as part of the psychometric evaluation.

**Table 1:** The Dysphagia Symptom Questionnaire (version 4.0) and score for each response option^a^

Question	Response options	Score
1. Since you woke up this morning, did you eat solid food?^b^	No	−
	Yes	−
2. Since you woke up this morning, has food gone down slowly or been stuck in your throat?	No	0
	Yes	2
3. For the most difficult time you had swallowing food today (during the past 24 hours), did you have to do anything to make the food go down or to get relief?	No, it got better or cleared up on its own	0
	Yes, I had to drink liquid to get relief	1
	Yes, I had to cough and/or gag to get relief	2
	Yes, I had to vomit to get relief	3
	Yes, I had to seek medical attention to get relief	4
4. The following question concerns the amount of pain you have experienced when swallowing food. What was the worst pain you had while swallowing food over the past 24 hours?^c^	None, I had no pain	0
	Mild	1
	Moderate	2
	Severe	3
	Very Severe	4

*DSQ* Dysphagia Symptom Questionnaire

^a^The scoring algorithm was constructed from responses to questions 2 and 3, to ensure that the final DSQ score was driven by the frequency and severity of dysphagia

^b^Responses to question 1 were unscored

^c^Responses to question 4 were not included as part of the psychometric analysis; question 4 is a standalone item on the DSQ

The DSQ scoring algorithm (Table 1) was therefore constructed from the responses to questions 2 and 3, to ensure that the final score was driven by the frequency and severity of dysphagia. To calculate the DSQ score, a minimum of eight diary entries were required for each 14-day period. Baseline DSQ scores were recorded during the 14-day period before randomization. DSQ scores could theoretically range from 0 to 84, with a lower score indicating less frequent or less severe dysphagia. The DSQ score was calculated using the following equation:

$$ DSQ\  score=\frac{\left( sum\  of points from questions\ 2\  and\ 3\  from daily\  DSQ\  diary\right)\times 14\  days}{number of diary days reported with non- missing data} $$

### Other physician- and patient-rated measures of disease activity

During the phase 2 trial, physicians were asked to complete the Physician Global Assessment (PGA) of disease activity (at the end of the baseline and treatment periods) using a visual analog scale (0–100 mm; ranging from no disease activity to worst possible disease activity) to provide a global assessment of EoE activity for each patient. Scores were categorized as follows: no disease to mild disease (0–30 mm); moderate disease (>30 to ≤70 mm); and worst possible disease (>70 to ≤100 mm). Patients were asked to complete the EoE Symptom Survey at the end of the baseline and treatment periods. The EoE Symptom Survey comprised a checklist of symptoms associated with EoE, and the options were phrased as follows: ‘heartburn (burning sensation in your chest)’; ‘chest pain (not heartburn or reflux related)’; ‘regurgitation (food coming back up into your mouth without vomiting/throwing up)’; ‘abdominal (stomach) pain’; ‘nausea (feeling sick to your stomach)’; and ‘vomiting (throwing up)’. Patients were asked to indicate, by ticking/checking, which symptoms they had experienced within the past 2 weeks. Patients were asked not to check symptoms that were unrelated to EoE. Patients were also asked to complete the Patient Global Impression of Change (PGIC) survey at the end of the treatment period, in which they were asked to rate the change in their dysphagia symptoms from baseline. Responses were: much better; better; a little better; no change; a little worse; worse; or much worse. EoE is a relatively newly described disease [1], and as such has not yet been formally assigned disease severity classifications. The above analyses are a first attempt to classify EoE disease severity using dysphagia symptoms.

### Stage 1: Item analysis

This stage of the analysis was used to examine the quality of completion (patient compliance with the DSQ), item distribution (floor and ceiling effects) and item discrimination. The choice of constructs was limited by the data that were collected for this analysis. Quality of completion was monitored to measure patient compliance. Item-level and patient-level missing data are reported; instances of missing data exceeding 10% during each 14-day period during baseline and treatment intervals were investigated for item-wise quality of completion. Item-level missing data were treated as a random effect for each of the measures and an additional pattern analysis conducted, to identify patterns of missing data. Patient-level missing data were used to record the proportion of patients with at least one missing item over the course of the study. Floor and ceiling effects were considered to be acceptable if the upper limit of missing data for randomized patients, for either the lowest or highest possible score for question 3, did not exceed 9%. To evaluate individual item performance on the DSQ, an item discrimination index was calculated by categorizing patient responses during the last 14 days of the baseline period into the lower (25th percentile) and higher (75th percentile) ranges of dysphagia frequency and severity. Based on the 25th and 75th percentile distributions of the DSQ domain score, patients were assigned into three groups: patients who had high (>75th percentile), moderate (25th to 75th percentile) or low (<25th percentile) DSQ scores. Patients’ actions to alleviate dysphagia symptoms (question 3) were also categorized into three groups: no action taken (answer option 1), moderate action taken (answer options 2–4) and extreme action taken (answer option 5). The proportion of patients in each of these three categories who endorsed each item was also recorded. The threshold for acceptability for item discrimination was set at >50% [13].

### Stage 2: Psychometric evaluation

This stage of the analysis was used to assess the construct validity, reliability and responsiveness of the DSQ. The subsequent results were used to provide recommendations for interpretability thresholds for minimal clinically important differences (MCIDs)/clinically important differences (CIDs).

### Construct validity

Construct (concurrent) validity was assessed to determine if items within the DSQ conformed to previous knowledge of relationships between dysphagia severity and other clinical measures. Concurrent validity was measured by assessing the correlation between DSQ score and scores from the PGA of disease activity, a conceptually similar outcome measure. An additional analysis was performed to examine the relationship between DSQ score and esophageal peak eosinophil count (eos/hpf). Correlation coefficient thresholds were set at between 0.4 and 0.7, and below 0.4 for the PGA of disease activity and eosinophil count analysis [14], respectively. The hypothesis was that the correlation between DSQ score and PGA of disease activity response would be moderate (>0.4). In contrast, correlation between DSQ score and peak eosinophil count (histologic response) was expected to be low (<0.4), owing to the potential time delay between histologic response and symptom improvement. These analyses were conducted using data collected from the last 14 days of the baseline period. For the known-groups method, DSQ scores were compared with categorical responses from the PGA of disease activity and from the EoE Symptom Survey. The EoE Symptom Survey was included to assess change in the other, less common symptoms of EoE over the 12-week treatment period.

### Test−retest reliability

The reliability of the DSQ was examined in patients who reported no change or minimal change in dysphagia symptoms on the PGIC survey. Patients were included if they completed the PGIC survey at the end of the treatment period (or earlier for patients who withdrew from the study) and recorded no change/minimal change in dysphagia symptoms. This analysis was performed using the intraclass correlation coefficient (ICC), calculated using Shrout–Fleiss reliability for a single score [14]. An ICC of 0.70 or above was considered acceptable [15].

### Responsiveness (ability to detect change)

Absolute change in DSQ score from baseline (visit 2) to subsequent treatment visits was expressed in terms of standardized effect size (SES), which was calculated based on Cohen’s recommendations [16]; and was designated as small (SES = 0.20), moderate (SES = 0.50) or large (SES = 0.80) [17]. Patients were placed into three categories based on changes in their DSQ scores: improvement; no change; and decline. The data were analyzed using the *χ* ^2^ test to determine the significance of the relationship between each pair of change variables. Paired *t*-tests and one-way analysis of variance were used to evaluate differences between groups. The relationship between histologic response and patient-reported change was subsequently assessed. Patients were categorized as either responders (≤6 eos/hpf) or non-responders (>6 eos/hpf) based on their histologic response at the end of the treatment period.

### Minimally important difference (meaningful change estimation)

Minimally important difference (MID) analyses were performed on the change in DSQ score from baseline (visit 2) to all available time points after baseline using distribution- and anchor-based methods. Distribution-based methods were used to estimate the MID based on the standard error of measurement. To calculate the standard error of measurement, the standard deviation (SD) of the DSQ score was multiplied by the square root of one minus its reliability coefficient (*r*):

$$ Standard\  err or of measurement= SD\times \sqrt{\Big(1}-r\Big) $$

Owing to the brevity of the scale, Cronbach’s alpha was not used for the reliability coefficient estimate. Rather, the Rasch person reliability, which is analogous to the Kuder–Richardson formula 20 [18], was used for each respective score.

Anchor-based methods were also used to determine the MCID and CID using absolute and percentage changes in mean DSQ score from baseline (visit 2). For this analysis, patients were grouped by PGIC score. The PGIC survey was selected to determine the anchor-based estimates for the MCID and CID because of the strong positive correlation observed between PGIC and DSQ scores (correlation coefficient >0.3) [19].

### Statistical analysis

Psychometric analyses were performed in SAS (version 9.3) (SAS Institute Inc., Cary, NC) and Winsteps (version 3.75.0) (Winsteps, Inc., Beaverton, OR). All analyses were pre-specified with distribution-based data transformation.

For stage 1 analyses: the quality of completion was assessed at all available time points by day and week for the baseline and treatment periods; item distribution was examined at all available time points by day and week for the baseline and treatment periods; and item discrimination was assessed using data collected during the last 2 weeks of the baseline period. For stage 2 analyses: concurrent validity was assessed using data from the final 2 weeks of the baseline period; known-groups validity was assessed using data collected from the final 2 weeks of the baseline period and until the end of the treatment period; test–retest reliability examined patients who completed the PGIC survey at the end of the treatment period (or earlier for patients who withdrew from the study) and recorded no change/minimal change in dysphagia symptoms; responsiveness was examined using the difference in scores from the baseline period to all subsequent treatment visits; and both MID analyses were conducted using the change in DSQ scores from baseline (14-day average) to all available post-baseline time points.

## Results

### Study population

Of the 119 patients who entered the baseline period, 93 proceeded to the 12-week treatment period and were randomized to receive BOS (*n* = 51) or placebo (*n* = 42). Patients included in both the baseline and treatment periods were considered for this analysis. Patient characteristics at baseline are shown in Table 2; these were similar between treatment arms [10]. Patients aged 11–40 years were enrolled; however, exemptions were granted for three patients (<11 years, *n* = 2, were considered mature enough to self-assess dysphagia and use the DSQ; >40 years, *n* = 1, no evidence of fibrostenotic disease and met all other inclusion criteria).

**Table 2:** Demographic and baseline characteristics for all randomized patients who entered the 12-week treatment period

Demographic variable	All patients	
Age, years (*n* = 93)		
Mean ± SD	21.6 ± 7.7	
Median (min, max)	21.0 (9, 42)	
≥18 years of age, *n* (%)	58 (62.4)	
Male, *n* (%) (*n* = 93)	64 (68.8)	
White, *n* (%) (*n* = 93)	88 (94.6)	
Height, cm (*n* = 93)		
Mean ± SD	172.3 ± 11.5	
Median (min, max)	174.1 (134.1, 195.6)	
Weight, kg (*n* = 93)		
Mean ± SD	70.1 ± 17.1	
Median (min, max)	(69.8, 117.1)	
Time since diagnosis of EoE, months^a^ (*n* = 87)		
Mean ± SD	37.6 ± 38.2	
Median (min, max)	26.3 (−0.1, 164.2)	
Physician Global Assessment of disease activity (*n* = 93)		
Mean ± SD	58.3 ± 21.5	
Median (min, max)	62.0 (0, 94.0)	
DSQ score (*n* = 92)		
Mean ± SD	29.8 ± 14.8	
Median (min, max)	28.0 (6.5, 61.1)	
Peak eosinophil count (eos/hpf)	Placebo (*n* = 42)	BOS (*n* = 51)
Mean ± SD	133.0 ± 81.6	157.8 ± 96.1

*BOS* budesonide oral suspension, *DSQ* Dysphagia Symptom Questionnaire, *EoE* eosinophilic esophagitis, *eos* eosinophils, *hpf* high-power field, *max* maximum, *min* minimum, *SD* standard deviation

^a^Defined as the number of days between the date of diagnosis and the screening visit date

### Stage 1: Item analysis

Item-level missing data were low for the baseline period, with 15.1% (197/1302) of item responses missing; however, this increased to 33.6% (219/651) by week 12 of the treatment period. In contrast, patient-level missing data were high, with 76.3% (71/93) of patients missing at least one item of the questionnaire across all visits during the baseline period (Table 3). This was the same at the end of the study (during week 12: 76.3%).

**Table 3:** Stage 1: Item analysis of the DSQ (version 4.0) to assess quality of completion by patients, item score patterns, item distribution and item discrimination at baseline (visit 2) in patients with EoE. Floor and ceiling effects at the end of the 12-week treatment period are also reported

Parameter	All patients (*n* = 93)		
*Quality of completion, % (n/N)*			
Missing data per item (questions 2 & 3)	15.1% (197/1302)		
Missing items per patient (questions 2 & 3)	76.3% (71/93)		
*Item score pattern* ^*a*^ *(n, number of responses)*	Total possible responses (*n* = 1302)		
Missing items	197		
No dysphagia (question 2: ‘No’)	301		
Dysphagia 1 (question 2: ‘Yes’; question 3: option 1)	238		
Dysphagia 2 (question 2: ‘Yes’; question 3: option 2)	401		
Dysphagia 3 (question 2: ‘Yes’; question 3: option 3)	133		
Dysphagia 4 (question 2: ‘Yes’; question 3: option 4)	31		
Dysphagia 5 (question 2: ‘Yes’; question 3: option 5)	1		
*Item distribution (question 3), % (n/N)*			
Floor effects at baseline	8.6% (8/93)		
Ceiling effects at baseline	6.5% (6/93)		
Floor effects at week 12	54.8% (46/84)		
Ceiling effects at week 12	8.3% (7/84)		
*Item discrimination* ^*b*^			
Question 2^c^	‘No dysphagia’	‘Dysphagia’	
High DSQ scores, *n* (%)	2 (1.9)	104 (98.1)	
Moderate DSQ scores, *n* (%)	33 (20.5)	128 (79.5)	
Low DSQ scores, *n* (%)	24 (28.6)	60 (71.4)	
Question 3^c^	‘No action’	‘Moderate action’	‘Extreme action’
High DSQ scores, *n* (%)	7 (6.6)	99 (93.4)	0 (0.0)
Moderate DSQ scores, *n* (%)	89 (55.3)	71 (44.1)	1 (0.6)
Low DSQ scores, *n* (%)	52 (61.9)	32 (38.1)	0 (0.0)

*DSQ* Dysphagia Symptom Questionnaire, *EoE* eosinophilic esophagitis

^a^Item scoring patterns defined as number of instances where a patient selected the responses indicated (question 2: ‘Yes’/‘No’; question 3: options 1–5) during the baseline period

^b^Item discrimination – based on the 25th and 75th percentiles of the DSQ domain score, patients were assigned into three groups: patients who had high (>75th percentile), moderate (25th to 75th percentile) and low (<25th percentile) DSQ scores. Patient action was defined as the attempt to alleviate symptoms as per answers to question 3: options 1–5 (no action taken = answer option 1, moderate action taken = answer options 2–4, extreme action taken = answer option 5)

^c^High DSQ scores, *N* = 106; moderate DSQ scores, *N* = 161; low DSQ scores, *N* = 84

Out of a possible 1302 responses, there were 1105 completed items and 197 missing items during the 14-day baseline period (visit 2). There were 804 instances where patients (*n* = 93) reported dysphagia (question 2: ‘Yes’), and 301 instances where patients reported no dysphagia (question 2: ‘No’). During this period, there were 238 instances where patients with dysphagia reported that it got better or cleared up on its own (question 2: ‘Yes’; question 3: response option 1); and 401 instances where patients with dysphagia reported that they had to drink liquid to get relief (question 2: ‘Yes’; question 3: response option 2). There were 133 and 31 instances where patients with dysphagia reported that they had to cough and/or gag or vomit, respectively in order to get relief (question 2: ‘Yes’; question 3: response option 3 or 4, respectively). Lastly, there was only one instance where a patient with dysphagia reported that they had sought medical attention to get relief (question 2: ‘Yes’; question 3: response option 5) (Table 3).

The DSQ measured the full range of dysphagia symptoms experienced by patients, with floor and ceiling effects below the pre-defined threshold (≤9%) at baseline (visit 2) (Table 3). However, by the end of the treatment period (week 12) the proportion of patients selecting the lowest DSQ scores had increased (54.8%); ceiling responses nonetheless remained below the threshold (8.3%). Overall, there was strong item discrimination among patients. Patients with more frequent dysphagia (question 2: ‘Yes’) (98.1%) during baseline (visit 2) reported within the highest (worst) range of DSQ scores (upper 25%) exceeding the >60% threshold for acceptability (Table 3). Similarly, patients who answered ‘Yes’ for question 2 were more likely to select the middle range of ‘symptom alleviation methods/moderate action’ (93.4%) for question 3, with only one patient selecting the highest category for this item (Table 3). Item characteristic curves at baseline (visit 2) showed a monotonically increasing function, with the steepness of the curve highlighting good item discrimination (data not shown).

### Stage 2: Psychometric evaluation

#### Construct validity

A weak but significant positive correlation was seen between DSQ score and physician-rated scores (PGA of disease activity), as determined by Spearman’s correlation test (correlation coefficient = 0.2587). This is below the predefined acceptability threshold (0.4–0.7). The known-groups method was also used to assess the construct validity of the DSQ, by comparing groups of patients who were categorized based on their EoE Symptom Survey and PGA of disease activity data (Table 4). At baseline (visit 2), patients with heartburn, chest pain, regurgitation, nausea or vomiting had significantly higher (worse) mean DSQ scores than patients without these symptoms (all *p* < 0.05). Similarly, at baseline (visit 2), patients who scored lowest (‘none to mild disease’) on the PGA of disease activity also had the lowest mean DSQ scores (Table 4). Patients’ DSQ scores then increased stepwise with increasing disease activity (‘moderate disease’ and ‘worst possible disease’) (Table 4). Combined, these data support the known-groups validity of the DSQ. An additional, exploratory analysis of histologic response and patient-reported change in DSQ score suggests a weak association (correlation coefficient = 0.0337), which removes this variable from consideration as a meaningful anchor in this evaluation.

**Table 4:** Stage 2: Psychometric evaluation of the DSQ (version 4.0) to assess the construct validity, concurrent validity, reliability and responsiveness of the DSQ in patients with EoE at baseline (visit 2)

Parameter	All patients (*n* = 93)
*Construct validity – known-groups method*	
EoE Symptom Survey, ^a^mean DSQ score	
Heartburn (− vs +)	26.6 vs 34.3 (*p* = 0.0140)
Chest pain (− vs +)	27.6 vs 34.1 (*p* = 0.0448)
Regurgitation (− vs +)	26.7 vs 34.9 (*p* = 0.0092)
Abdominal pain (− vs +)	28.7 vs 31.8 (*p* = 0.3476)
Nausea (− vs +)	27.7 vs 34.9 (*p* = 0.0325)
Vomiting (− vs +)	26.9 vs 49.2 (*p* < 0.0001)
*Concurrent validity – PGA of disease activity*, ^b^mean DSQ score	21.1 vs 29.5 vs 33.7 (*p* = 0.0709)
*Test−retest reliability* ^*c*^	ICC = 0.82
*Responsiveness*, ^d^mean DSQ score	
Responder (≤6 eos/hpf)	−16.2 (14.3) [−1.09], *p* < 0.0001
Non-responder (>6 eos/hpf)	−9.9 (11.6) [−0.67], *p* < 0.0001

*ANOVA* analysis of variance, *DSQ* Dysphagia Symptom Questionnaire, *EoE* eosinophilic esophagitis, *eos* eosinophils, *hpf* high-power field, *ICC* intra-class correlation coefficient, *PGA* Physician Global Assessment, *SES* standardized effect size

^a^Comparison of mean DSQ scores for patients with (+) or without (−) symptom (*p* value, paired *t*-test): heartburn, − (*n* = 54) + (*n* = 38); chest pain, − (*n* = 61) + (*n* = 31); regurgitation, − (*n* = 57) + (*n* = 35); abdominal pain, − (*n* = 61) + (*n* = 31); nausea, − (*n* = 65) + (*n* = 27); vomiting, − (*n* = 80) + (*n* = 12)

^b^Comparison of mean DSQ scores for patients with none to mild (*n* = 10), moderate (*n* = 56) or worst possible disease (*n* = 26) (*p* value, one-way ANOVA)

^c^Test–retest reliability, *n* = 93

^d^Responsiveness defined as mean change in DSQ score from baseline (visit 2), mean change (SD) [SES], *p* value (responders [≤6 eos/hpf], *n* = 20; non-responders [>6 eos/hpf], *n* = 67)

### Test−retest reliability

The test−retest reliability of the DSQ was measured to assess the stability of DSQ scores over time (when no change was expected), i.e. in those who reported no change or minimal changes on the PGIC survey from baseline (visit 2) to week 12 (or earlier). The ICC was 0.82, which met the threshold for acceptability (≥0.70), supporting the reliability of the DSQ (Table 4).

### Responsiveness (ability to detect change)

Overall, there was a decrease in mean DSQ score from baseline (visit 2) to the end of the 12-week treatment period, and this correlated with histologic response (Table 4). Patients with a histologic response (≤6 eos/hpf) exhibited a greater improvement in DSQ scores from baseline (visit 2): mean change (±SD) [SES]: −16.2 (±14.3) [−1.09]; *p* < 0.0001 than those who did not have a histologic response (>6 eos/hpf): mean change: −9.9 (±11.6) [−0.67]; *p* < 0.0001 (Table 4).

### Minimally important differences (meaningful change estimation)

Absolute and percentage distribution-based MIDs in DSQ score were estimated at 7.4 points and 8.9%, respectively (Table 5), and were driven by the overall distribution of DSQ scores at baseline (visit 2). Most (74.4% [67/90]) patients who reported an improvement using the PGIC survey (‘a little better’, ‘better’ and ‘much better’) also reported a concomitant improvement in dysphagia symptoms (lower DSQ scores) (Table 5). Using the anchor-based methods, the MCID and CID in DSQ score (mean absolute change) were estimated to be −6.5 points (‘a little better’) and −13.5 points (‘better’), respectively. The MCID and CID in DSQ score (mean percentage change) were estimated to be −27.4% (‘a little better’) and −55.4% (‘better’), respectively (Table 5).

**Table 5:** Estimates of meaningful change in DSQ score in patients with EoE at baseline and from baseline to week 12 of the treatment period

	Mean baseline DSQ score (absolute)				Mean baseline DSQ score (percentage)			
Distribution-based^a^	*n*	Mean	SD	½ SD	*n*	Mean (%)	SD	½ SD
	93	29.9	14.8	7.4	93	35.4	17.7	8.9
	Mean change in DSQ score (absolute)				Mean change in DSQ score (percentage)			
Anchor-based^b^	*n*	Mean change	SD	½ SD	*n*	Mean change (%)	SD	½ SD
Much better	20	−20.0	14.5	–	20	−75.4	25.1	–
Better^c^	23	−13.5	12.7	–	23	−55.4	41.2	–
A little better^d^	24	−6.5	8.1	–	24	−27.4	43.6	–
No change	14	−5.4	9.0	–	14	−15.4	37.6	–
A little worse	6	−3.6	7.7	–	6	−12.7	49.7	–
Worse	2	−1.6	4.0	–	2	−2.8	8.0	–
Much worse	1	28.0	–	–	1	100.0	–	–

*CID* clinically important difference, *DSQ* Dysphagia Symptom Questionnaire, *EoE* eosinophilic esophagitis, *MCID* minimal clinically important difference, *MID* minimally important difference, *PGIC* Patient Global Impression of Change, *SD* standard deviation

^a^Distribution-based estimates were driven by the overall distribution of DSQ scores at baseline (visit 2)

^b^Anchor-based estimates were driven by the mean change in DSQ scores from baseline (visit 2) to week 12 in patients by their PGIC groupings

^c^Estimate for CID

^d^Estimate for MCID

## Discussion

EoE is a chronic disease characterized by eosinophil-induced inflammation and symptoms of esophageal dysfunction, including dysphagia [1, 2]. EoE is a relatively newly described disease [1], and as such, has not yet been assigned disease severity classifications. There are currently no approved drugs indicated for EoE; however, treatment with a newly developed formulation of budesonide (BOS) has been shown to result in histologic improvements in pediatric patients [9] and histologic, endoscopic and symptom improvements in adolescents and adults with this disease [10, 20]. The primary objective of this analysis was to assess the psychometric properties of the DSQ, a PRO measure of dysphagia in patients with EoE, using data collected from a 12-week, phase 2, multicenter, randomized, double-blind, placebo-controlled trial of BOS in adolescents and adults with EoE [10].

Item characteristics showed that the DSQ measured the full range of dysphagia symptoms experienced by patients at baseline, with floor and ceiling effects below the predefined threshold, reflecting a high level of sensitivity. The proportion of patients with the lowest DSQ scores increased by week 12, indicative of an improvement in symptoms over time for all patients. Results from the DSQ were also consistent with data collected from a range of physician- and other patient-reported outcome measures (PGA of disease activity, EoE Symptom Survey and PGIC), providing evidence of good construct validity. The test–retest analysis highlighted that DSQ scores were consistent over time for patients who reported no change or minimal change in dysphagia symptoms. Similarly, patients who had a histologic response (≤6 eos/hpf) also reported a greater improvement in DSQ score (large SES; ≥0.80), compared with those who had a weaker histologic response (>6 eos/hpf; moderate SES; ≥0.50 to <0.80). These findings support the DSQ as a reliable measure of dysphagia in adolescents and adults with EoE.

A weak but positive association was observed between the change in DSQ score and peak eosinophil counts in the esophageal mucosa at the end of treatment. This was not unexpected; a number of previous studies of EoE have reported a lack of correlation between histologic findings and symptoms [9, 21, 22]. This may be explained by a potential time delay between histologic improvement and symptom improvement after treatment [12]. In addition, the magnitude of improvement in eosinophil density from baseline was not used for this analysis. Instead, the final absolute peak eosinophil count was described using the >6 eos/hpf and ≤6 eos/hpf cut-offs at the end of treatment. This may have also contributed to the weak association observed between change in DSQ score and peak eosinophil counts at the end of the study.

Using distribution-based methods, the absolute and percentage MID in DSQ score were estimated at 7.4 points and 8.9%, respectively. Using anchor-based methods, the MCID and CID in DSQ score (absolute change) were estimated to be −6.5 points and −13.5 points, respectively. The MCID and CID (percentage change) in DSQ score were estimated to be −27.4% and −55.4%, respectively. Percentage change in MCID and CID enable the comparison of data from different studies, in which baseline DSQ scores for the study populations may differ. These findings support the use of distribution- and anchor-based methods for the psychometric evaluation of the DSQ.

Despite the increasing prevalence of EoE worldwide [23], there have been very few randomized, controlled studies examining this disease [24–27]. The development of PRO measures for use in future studies of EoE would assist in the evaluation of new therapies. A number of other Clinical Outcome Assessments (COAs) are available or in development, including the Mayo Dysphagia Questionnaire-30 Day (MDQ-30) [28], the Pediatric EoE Symptom Score (PEESS) [22, 29], the Pediatric Quality of Life Inventory (PedsQL) [30] with the EoE-specific disease module (PedsQL EoE) [31], the Esophageal Symptom Questionnaire (ESQ) [32] and the Adult EoE Quality of Life questionnaire (EoE-QoL-A) [33]. However, the MDQ-30, to our knowledge, has not been validated among patients with EoE [12] and the other COAs specifically target either children or adults, respectively, and focus on a range of symptoms. The DSQ (version 4.0) therefore represents the first daily symptom diary to undergo structured psychometric validation specifically for EoE in both adolescents and adults with this disease.

The DSQ (version 4.0) has several strengths over other tools. First, the DSQ was designed and evaluated in accordance with current US FDA guidelines [12, 34], with the aim of developing a valid symptom measure for use in clinical trials. The DSQ also uses a daily recall period, allowing patients to remember and record their symptoms more clearly, rather than relying on longer recall periods. This short recall period was previously assessed and considered appropriate for this disease [12]. A short recall period is particularly useful for diseases in which symptoms can fluctuate [35]. Lastly, the DSQ is a disease-specific symptom measure developed to assess the most common symptom of EoE experienced by adult patients – dysphagia [5, 23], while other PRO measures attempt to consider a broader range of symptoms for EoE [9, 22]. Dysphagia was selected as the primary symptom measure based on a literature review, previous trials, existing symptom instruments, expert opinion and patient interviews, as part of the conceptual framework development (content validation) [12]. More recently, clinical features (e.g. peak eosinophil counts and eosinophil peroxidase levels) and gene expression profiles associated with EoE have been shown to correlate most strongly with dysphagia, as opposed to other symptoms, such as nausea/vomiting or pain [36].

With regard to limitations, levels of non-compliance with the DSQ (missing data) increased towards the end of the study (week 12); however, attempts were made to limit this by excluding patients who were highly non-compliant during the screening period. In addition, alerts were sent, in a blinded fashion, to center personnel at sites where consecutive non-compliance events were detected. Owing to the high level of patients missing at least one day during the data collection weeks, patient training should be enhanced in order to increase DSQ compliance rates for future studies. The handling of item-level missing data is a focus of statistical consideration in clinical trial analyses; in this study, item-level missing data were treated as a random effect for each of the measures and an additional pattern analysis was conducted, to account for this effect. The lack of reliable, objective clinical anchors for EoE was another limitation, which could have potentially introduced variation [37]; however, this analysis used recognized patient- and physician-rated measures of disease severity (PGIC and PGA, respectively) as anchors in this study. Lastly, in establishing patient selection and eligibility criteria for this randomized controlled trial, the randomized population may not represent the entire EoE population. Nevertheless, this group had similar demographic characteristics and the principle EoE symptom of dysphagia to those from a natural history study and are therefore representative of those real-life patients who may seek therapeutic treatment for their EoE symptoms [38].

## Conclusions

The DSQ was able to reliably discriminate between levels of disease severity in patients with EoE, using dysphagia as the primary symptom measure. The DSQ also offered an acceptable level of agreement between clinician, patient and histologic responses. In summary, the DSQ has been shown to have good construct validity and to reliably measure frequency and severity of dysphagia. The DSQ should therefore be considered as a viable PRO measure for use in future therapeutic studies of EoE.

## Abbreviations

BOS: Budesonide oral suspension
CID: Clinically important difference
COA: Clinical Outcome Assessment
DSQ: Dysphagia Symptom Questionnaire
EoE Symptom Survey: Eosinophilic Esophagitis Symptom Survey
EoE-QoL-A: Adult Eosinophilic Esophagitis Quality of Life questionnaire
ESQ: Esophageal Symptom Questionnaire
FDA: Food and Drug Administration
hpf: High-power field
ICC: Intra-class correlation coefficient
MCID: Minimal clinically important difference
MDQ-30: Mayo Dysphagia Questionnaire-30 Day
MID: Minimally important difference
PedsQL: Pediatric Quality of Life Inventory
PedsQL EoE: Pediatric Quality of Life Inventory with the Eosinophilic Esophagitis-specific disease module
PEESS: Pediatric Eosinophilic Esophagitis Symptom Score
PGA: Physician Global Assessment
PGIC: Patient Global Impression of Change
PRO: Patient-reported outcome
SD: Standard deviation
SES: Standardized effect size
